# Multi-Omics Reveals Inhibitory Effect of Baicalein on Non-Alcoholic Fatty Liver Disease in Mice

**DOI:** 10.3389/fphar.2022.925349

**Published:** 2022-06-15

**Authors:** Ping Li, Jianran Hu, Hongmei Zhao, Jing Feng, Baofeng Chai

**Affiliations:** ^1^ Institute of Loess Plateau, Shanxi University, Taiyuan, China; ^2^ Department of Biological Science and Technology, Jinzhong University, Jinzhong, China; ^3^ Department of Gastroenterology, Shanxi Provincial People’s Hospital Affiliated to Shanxi Medical University, Taiyuan, China

**Keywords:** baicalein, gut microbiota, transcriptiomic profiling, metabolomic profiling, integrated analysis

## Abstract

Non-alcoholic fatty liver disease (NAFLD) is the most common chronic liver disease, whose etiology is poorly understood. Accumulating evidence indicates that gut microbiota plays an important role in the occurrence and progression of various human diseases, including NAFLD. In this study, NAFLD mouse models were established by feeding a high-fat diet (HFD). Baicalein, a natural flavonoid with multiple biological activities, was administered by gavage, and its protective effect on NAFLD was analyzed by histopathological and blood factor analysis. Gut microbiota analysis demonstrated that baicalein could remodel the overall structure of the gut microbiota from NAFLD model mice, especially *Anaerotruncus*, *Lachnoclostridium,* and *Mucispirillum.* Transcriptomic analysis showed baicalein restored the expressions of numerous genes that were upregulated in hepatocytes of NAFLD mice, such as *Apoa4*, *Pla2g12a*, *Elovl7*, *Slc27a4*, *Hilpda*, *Fabp4*, *Vldlr*, *Gpld1*, and *Apom*. Metabolomics analysis proved that baicalein mainly regulated the processes associated with lipid metabolism, such as alpha-Linolenic acid, 2-Oxocarboxylic acid, Pantothenate and CoA biosynthesis, and bile secretion. Multi-omics analysis revealed that numerous genes regulated by baicalein were significantly correlated with pathways related to lipid metabolism and biosynthesis and secrection of bile acid, and baicalein might affect lipid metabolism in liver *via* regulating the ecological structure of gut microbiota in NAFLD mice. Our results elucidated the correlated network among diet, gut microbiota, metabolomic, and transcriptional profiling in the liver. This knowledge may help explore novel therapeutic approaches against NAFLD.

## Introduction

Non-alcoholic fatty liver disease (NAFLD) is a common liver disease worldwide. NAFLD affects both children and adults because of the dramatic rise in the prevalence of obesity, diabetes (Vuppalanchi and Chalasani, 2009; Majumdar et al., 2021), hypertension (Ryoo et al., 2014), and dyslipidemia (Katsiki et al., 2016). Between 2009 and 2019, the incidence rate of liver complications related to NAFLD increased in most Asian countries. In 2019, there were 170,000 incident cases and 168,959 deaths worldwide. Of these, 48.3% of the incident cases and 46.2% of the deaths occurred in Asia (Golabi et al., 2021). Recent research has demonstrated ingestion of high fat and intestinal dysbiosis may trigger NAFLD (Lambertz et al., 2017). Excess dietary fats might induce the accumulation of non-esterified fatty acids and then result in potential lipotoxins (Ferramosca and Zara, 2014).

Increasing evidence shows that disturbances in the gut microbiota may result in liver diseases including NAFLD (Tokuhara, 2021), since nutrients and microbiota-related components transfer from the intestines to the liver directly through the portal tract (Chen et al., 2019; Hong et al., 2019; Suk and Kim, 2019; Yuan et al., 2019). Gut microbiota has been proved to modulate a variety of physiological processes, such as the digestion of dietary fiber, the absorption of monosaccharides, the secretion of glucagon-like peptide-1, the suppression of bile acid production, and inflammation (Doulberis et al., 2012). The excess sugar and lipids associated with hepatic steatosis could be regulated by gut microbiota (Gkolfakis et al., 2015). Therefore, gut microbiota could be the potential therapeutic target to alleviate NAFLD.

Some natural compounds such as coffee (and its components), tormentic acid, verbascoside, and silymarin showed protective effects in ameliorating the critical pathological events involved in NAFLD. For example, silymarin has been widely used in the treatment of various liver disorders because of its hepatoprotective properties, including anti-inflammatory, antiproliferative, immunomodulatory, and nticholesterolemic properties (Salvoza et al., 2022). Baicalein, a bioactive flavone, is isolated from *Scutellariae baicalensis*, which is a traditional Chinese herb. Growing evidence shows that baicalein has a variety of pharmacological activities, such as anti-inflammatory, anticancer, and anti-oxidant effect (Pu et al., 2012; Li et al., 2020). Baicalein was also proved to be a promising compound for NAFLD and could be used as a dietary supplement to reduce hepatic fat accumulation and to ameliorate NAFLD-related biochemical abnormalities. Hepatic lysosomal acidification may be the potential target of baicalein (Zhu et al., 2020), and the mTOR pathway (Zhu et al., 2020), the AMPK pathway, SREBP1 signaling, and the synthesis of hepatic fat were proved to be affected by bacalein (Sun et al., 2020). Thus, the mechanism of baicalein against NAFLD might involve multiple targets and pathways.

Here, we identified the effects of baicalein ameliorating NAFLD. To explore the mechanism systematically, transcriptomics and metabolomics in the liver and metagenomics analysis in the high-fat diet (HFD)-fed mouse model (C57BL/6n) were combined, and bioinformatics analysis was carried out. Overall, these results should contribute new insight into the molecular mechanism of baicalein against NAFLD and suggest specific targets and pathways for the development of novel treatments that have the same beneficial effects.

## Methods and Materials

### Mice and Reagent

Healthy male C57BL/6N mice (15–20 g, 4 weeks old) were purchased from Beijing Vital River Laboratory Animal Technology Co., Ltd. All animal experiments were strictly in accordance with published National Institutes of Health guidelines and approved by the Committee on the Ethics of Scientific Research of Shanxi Medical University (Shanxi, China). The mice were randomized into five groups (*n* = 10): 1) Control (C, gavaged with equal volume saline), 2) Model (M, gavaged with equal volume saline), 3) Positive (P, gavaged with silymarin 200 mg/kg), 4) baicalein with high concentration (H, gavaged with baicalein 200 mg/kg/day), and 5) baicalein with low concentration (L, gavaged with baicalein 100 mg/kg/day). The C group was administered a normal basal diet and drinking water, whereas all the other groups were fed purified diets containing 60 kcal% (Research Diets, D12492, high fat diet; HFD) for 5 weeks. Body weights were measured weekly. The levels of blood glucose and insulin were determined after 12 h of fasting using an ELISA kit (Nanjing Jiancheng, Nanjing, China), respectively. At experiment completion, all mice were terminated by cervical dislocation. Blood was collected, and plasma was obtained after centrifugation (4°C; 1,500 × g, 10 min) and stored at −20°C. Liver tissue for RNA extraction and metabolic analysis and luminal contents of the cecum were flash-frozen in liquid nitrogen and stored at −80°C.

Baicalein, 5,6,7-Trihydroxyflavone (C_15_H_10_O_5_, HPLC > 98%), was purchased from Sigma-Aldrich (St. Louis, MO, United States).

### Histopathological Analysis

The paraffin-embedded liver tissue was cut into 4-μm-thick sections that were stained with hematoxylin and eosin (H&E). All stained liver slides were observed using a light microscope and evaluated for the NAFLD activity score (NAS), according to the previous report (Kleiner et al., 2005). Briefly, hepatic steatosis, lobular inflammation, and hepatic ballooning were investigated. In brief, steatosis was scored on a scale of 0–3 according to the following criteria: 0 (<5%), 1 (5%–33%), 2 (33%–66%), or 3 (>66%). Lobular inflammation was scored on a scale of 0–3 according to the following criteria: 0 (No foci), 1 (<2 foci per 20× optical field), 2 (2–4 foci per 20× optical field), or 3 (>4 foci per 20× optical field). Hepatocellular ballooning was scored on a scale of 0–2 according to the following criteria: 0 (none), 1 (mild, few), or 2 (moderate, many). The levels of TC, TG, LDL-C, HDL-C, ALT, and AST were detected using commercial kits (Nanjing Jiancheng, Nanjing, China).

### Transcriptomic Analysis and Real-Time PCR

Total RNA was extracted from mouse liver tissue using TRIzol agent according to the manufacturer’s protocols. RNA was further quantified using agarose gel electrophoresis, a NanoPhotometer spectrophotometer, and an Agilent 2100 bioanalyzer. Oligo (dT) magnetic beads were used to enrich the total RNA with Poly A structure. RNA seq libraries were generated using a NEBNext® UltraTM RNA Library Prep Kit for Illumina® (NEB, United States). The library quality was assessed on the Agilent Bioanalyzer 2100 system. Sequencing was performed by generating 50 base reads on an Illumina HiSeq platform (Illumina). Clean data were obtained by removing reads containing adapters, reads containing poly-N and low-quality reads from raw data. The paired-end clean reads were aligned to the reference genome using Hisat2 v2.0.5. The original readcount is normalized, mainly to correct the sequencing depth. Differential expression analysis was performed using the DESeq2 R package (1.16.1). *p*-values were adjusted using Benjamini and Hochberg’s approach for controlling the false discovery rate. Genes with |log2(FoldChange)| > 0 and Padj < 0.05 found using DESeq2 were assigned as differentially expressed. The FDR value was obtained by multiple hypothesis test correction. Gene Ontology (GO) and KEGG (Kyoto Encyclopedia of Genes and Genomes) enrichment analysis were determined using the clusterProfiler R package.

A High Capacity cDNA Reverse Transcription Kit (Applied Biosystems, United States) was used to synthesize cDNA. The real-time PCR was conducted using the SYBR@Premix Ex Taq™ (Perfect Real Time) (Takara, China) by the StepOne Real-Time PCR system (Thermo Fisher Scientific, United States). The primers are listed in Supplementary Table S1. The results are presented as the means ± standard deviation (SD). Statistical analysis was performed using GraphPad Prism 5.0 (GraphPad, San Diego, CA, United States). Student’s two-tailed *t*-test was used as appropriate. *p*-value < 0.05 was considered statistically significant.

### Metabolomic Analysis

Liver tissues (100 mg) were individually grounded with liquid nitrogen, and the homogenate was resuspended with prechilled 80% methanol and 0.1% formic acid by well vortexing. The samples were incubated on ice for 5 min and were then centrifuged at 15,000 rpm and 4°C for 5 min. Supernatant was diluted to the final concentration containing 60% methanol by LC-MS grade water. The samples were subsequently transferred to a fresh Eppendorf tube with a 0.22-μm filter and were then centrifuged at 15,000 g and 4°C for 10 min. LC-MS/MS analyses were performed using a Vanquish UHPLC system (Thermo Fisher) coupled with an Orbitrap Q Exactive series mass spectrometer (Thermo Fisher).

These metabolites were annotated using the KEGG database, HMDB database, and Lipidmaps database. Principal components analysis (PCA) and partial least squares discriminant analysis (PLS-DA) were performed at metaX (Wen et al., 2017). Univariate analysis (*t*-test) was used to calculate the *p* value. The metabolites with VIP > 1 and *p*-value< 0.05 and fold change≥ 2 or FC≤ 0.5 were considered to be differential metabolites. Volcano plots were used to filter metabolites of interest which were based on Log2 (FC) and −log10 (*p*-value) of metabolites.

### Gut Microbiota Analysis

Total genome DNA from luminal content of the cecum was extracted using the CTAB/SDS method. The 16S rRNA V3-V4 region was amplified using a specific primer (16S V4: 515F-806R) with the barcode. Sequencing libraries were generated using the Ion Plus Fragment Library Kit (Thermo Scientific, United States) following the manufacturer’s recommendations. The library quality was assessed on a Qubit@ 2.0 Fluorometer (Thermo Scientific). At last, the library was sequenced on an Ion S5TM XL platform. The clean reads were obtained by using the Cutadapt and UCHIME algorithm. Sequences analysis was performed using Uparse software (Uparse v7.0.1001, http://drive5.com/uparse/). To investigate the phylogenetic relationship of different OTUs and the difference of the dominant species in different groups, multiple sequence alignment was conducted using MUSCLE software. Alpha diversity and beta diversity were calculated using QIIME software (Version 1.7.0). Principal component analysis (PCA), UniFrac distance-based non-metric multidimensional scaling (NMDS), and the unweighted pair-group method with arithmetic means (UPGMA) were performed using R software. Tax4Fun functional prediction was achieved using the nearest neighbor method based on the minimum 16S rRNA sequence similarity by extracting the KEGG database prokaryotic whole genome 16S rRNA gene sequence and aligning it to the SILVA SSU Ref NR database using the BLASTN algorithm (BLAST Bitscore > 1500) to establish a correlation matrix and map the prokaryotic whole genome functional information of the KEGG database annotated by UProC and PAUDA to the SILVA database to implement the SILVA database function annotation.

### Integrative Analysis of Multi-Omics Data

Correlation between the top 100 DEGs and top 50 differential metabolites was analyzed using the Pearson statistical method, and the correlation coefficient R2 and *p* value were calculated. Red color indicates the positive correlation, and the blue color means the negative correlation.

The Pearson statistical method was used to access the correlation between the top 20 differential bacteria and top 10 differential metabolites. The correlation coefficients rho (∣rho∣≥ 0.8) and *p* value (*p* ≤ 0.05) were calculated. Red color indicates the positive correlation, and the blue color means the negative correlation. The flatness of the ellipse represents the absolute value of the correlation.

## Results

### Hepatoprotective Effect of Baicalein on High-Fat Diet–Induced Non-Alcoholic Fatty Liver Disease in Mice

**FIGURE 1:**
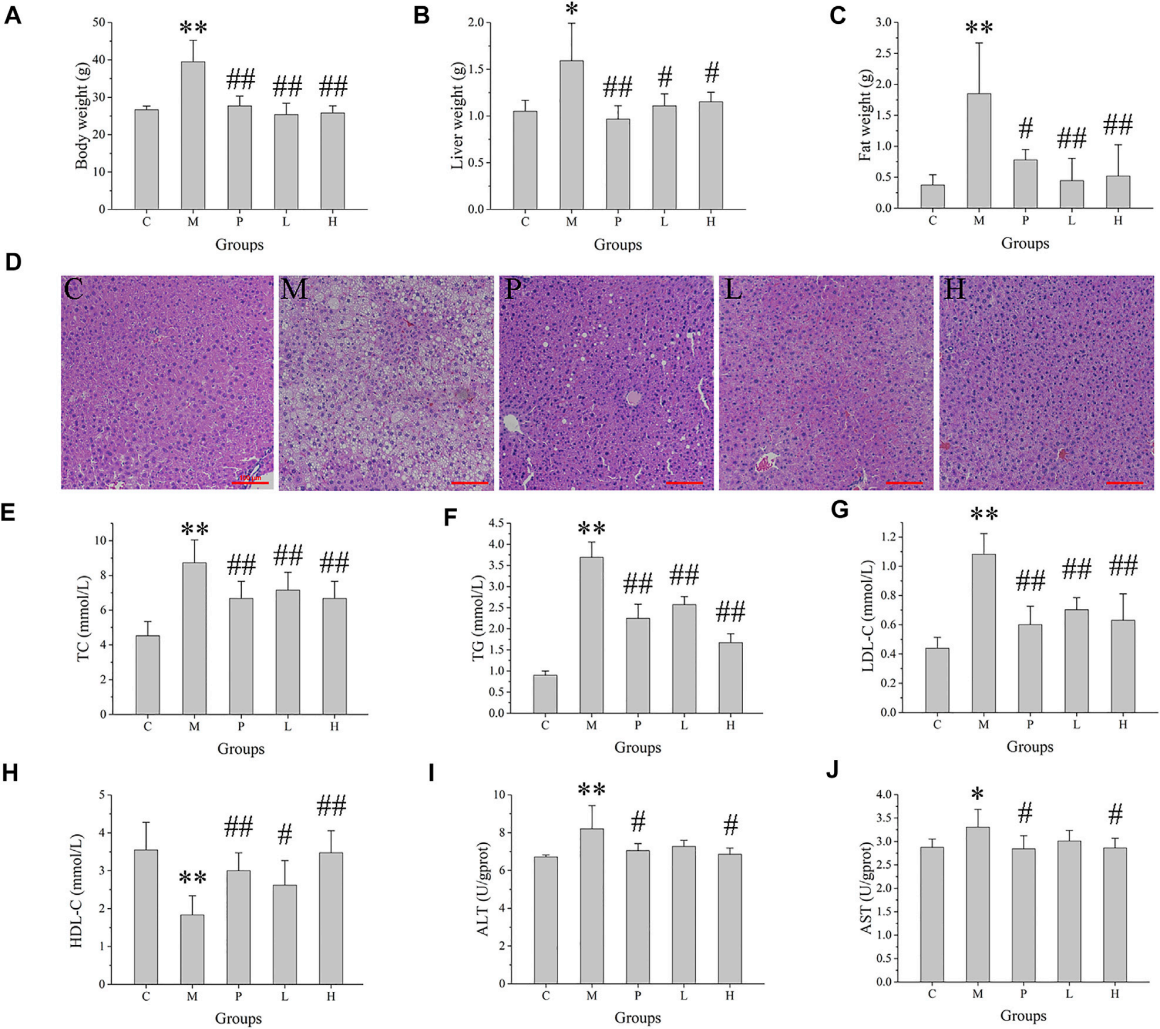
Effects of baicalein treatment on the liver and lipid metabolism. **(A)** Body weight. **(B)** Liver weight. **(C)** Epididymal fat weight. **(D)** Histopathological analysis of liver. **(E)** Serum total cholesterol (TC). **(F)** Serum total triglyceride (TG). **(G)** Serum low density lipoprotein-cholesterol (LDL-C). **(H)** Serum high density lipoprotein-cholesterol (HDL-C). **(I)** Serum alanine aminotransferase (ALT) activity. **(J)** Serum aspartate aminotransferase (AST) activity. Data are presented as mean ± SD (*n* = 10). **p < 0.01, *p < 0.05, compared with the C group; ##*p* < 0.01, #*p* < 0.05, compared with the M group.

To access the protective effect of baicalein on NAFLD, body weight, liver weight, and epididymal fat weight were determined. There was no significant difference in the initial body weight of each group. The mice fed with high-fat diet (M group) continuously for 5 weeks showed a clear increase in body weight (*p* < 0.01), compared with the control groups (fed with normal basal diet) (Figure 1A). Administration of silymarin (P group) or baicalein (L and H group) clearly decreased the body weight (*p* < 0.01), compared with the M group. In addition, the liver weight and the epididymal fat weight in NAFLD mice (M group) were elevated (*p* < 0.01) by 3.4- and 4.9-fold, relative to the control mice, respectively, and treatment of silymarin (P group) or baicalein (L and H group) could significantly decrease this HFD-induced increase in liver weight and epididymal fat weight (Figures 1B,C). Pathological examination of liver tissues revealed frequent incidence of macrosteatosis and hepatocyte ballooning in NAFLD mice, which was ameliorated in livers of silymarin- and baicalein-treated mice (Figure 1D). Compared with the C group, the liver tissue of mice in the M group obtained higher scores of steatosis, hepatocyte ballooning, lobular inflammation, and NAS score, indicating that the NAFLD model was successfully established. Compared with the M group, the scores of the P group decreased significantly, while the scores of the L and H groups were lower (Supplementary Figure S1A). Additionally, compared with the C group, the fasting blood glucose (*p* < 0.01) and insulin (*p* < 0.01) were extremely high in the M group and decreased in silymarin- and baicalein-treated mice (P, L, and H groups, *p* < 0.01) (Supplementary Figures S1B,C). After all mice were sacrificed, several blood factors were determined by ELISA assay. As shown in Figures 1E–J, NAFLD mice exhibited higher serum TC, TG, LDL-C, ALT, and AST and lower serum HDL-C, indicating metabolic disorders and NAFLD symptoms. After treatment with silymarin (P group) or baicalein (L and H groups), the levels of serum TC, TG, LDL-C, ALT, and AST decreased significantly, while the levels of HDL-C increased (*p* < 0.01). Therefore, silymarin and baicalein could both improve the disorder of lipid metabolism in NAFLD mice, while baicalein exhibited a better effect.

### Variations of Global Transcriptional Profiling in Liver of Non-Alcoholic Fatty Liver Disease Mice

**FIGURE 2:**
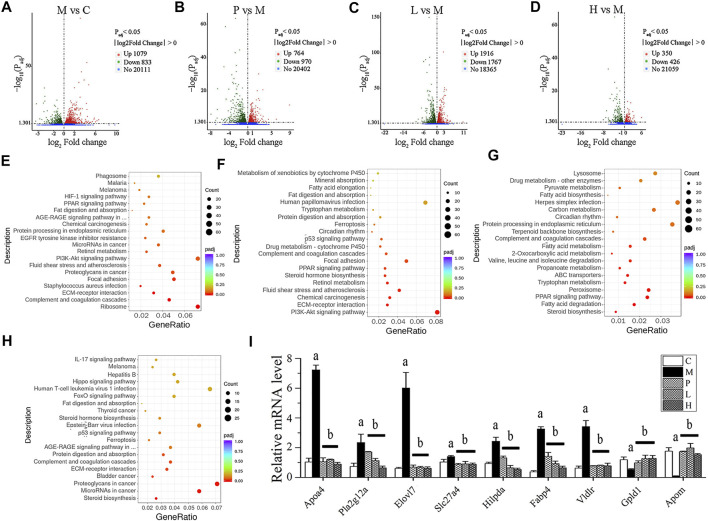
Transcriptomic analysis of liver tissue. DEGs were displayed in volcano plot graphs [**(A)** M vs. C; **(B)** P vs. M; **(C)** L vs. M; and **(D)** H vs. M] (*n* = 3). The DEGs were enriched in the KEGG pathway database [**(E)** M vs. C; **(F)** P vs. M; **(G)** L vs. M; and **(H)** H vs. M] (*n* = 3). **(I)** mRNA expression in livers was detected by real-time PCR. **(A)** *p* < 0.01, compared with the C group; **(B)** *p* < 0.01, compared with the M group.

Transcriptomic analysis was conducted on the mRNA from the livers of C, M, P, L, and H groups. The DEGs were illustrated in a heatmap (Supplementary Figure S2A), and the overlapped ones from three comparisons (P vs. M, L vs. M, and H vs. M) were shown in a Venn diagram (Supplementary Figure S2B). Compared with the C group, the M group had 1,912 DEGs (Supplementary Table S2), of which 1,079 were upregulated (Figure 2A; Supplementary Table S3), and 833 were downregulated (Figure 2A; Supplementary Table S4). After the treatment of silymarin, 1,734 DEGs were found (Supplementary Table S2), including 764 upregulated (Figure 2B; Supplementary Table S5) and 970 downregulated (Figure 2B; Supplementary Table S6), of which 214 transcripts were restored, with regard to the expression level of the C group (Supplementary Table S7). Similarly, compared with the M group, 3,683 DEGs with∣log2fold change∣≥1 and Padj<0.05 were screened in the L group (Supplementary Table S2), presenting as volcano plot graphs (Figure 2C). 1,916 of them were upregulated (Supplementary Table S8) and 1,767 of them were downregulated (Supplementary Table S9), and expressions of 46 transcripts of them were similar to those in the C group (Supplementary Table S10). Additionally, DEGs containing 350 upregulated (Supplementary Table S11 and 426 downregulated (Supplementary Table S12) were revealed in the H group vs. the M group (Figure 2D), and the expression of 21 of them were similar to those in the C group (Supplementary Table S13). 640 of the DEGs in the L group vs. the M group and the H group vs. the M group were overlapped (Supplementary Figure S2B; Supplementary Table S14), and 278 of them were positively regulated by baicalein (Supplementary Table S15), while 362 were negatively regulated by baicalein (Supplementary Table S16). Therefore, these 640 transcripts might play an important role in the process of baicalein against NAFLD. Notably, 476 of these 640 transcripts also showed consistent alterations under silymarin treatment (Supplementary Figure S2B) and number 1–476 in Supplementary Table S16), indicating that these transcripts might be the common critical genes of silymarin and baicalein in alleviating NAFLD symptoms.

To further investigate characterization of DEGs, we performed enrichment analysis using the GO and KEGG pathway database. Supplementary Figures S3A,D depicts the enriched biological processes (BPs), cellular components (CCs), and molecular functions (MFs) of the targets, which are mainly associated with response to baicalein. As shown in Figure 2E, the DEGs in the M group vs. the C group involved in the BPs related to angiogenesis, cell movement, extracellular matrix organization, and so on; the coding products of DEGs were mainly distributed in CCs, including the ribosome, extracellular matrix, cytosolic part, and basement membrane; as to the enriched MFs, the target proteins were mainly connected with structural constituent of ribosome, structural molecule activity, glycosaminoglycan binding, growth factor binding, heparin binding, sulfur compound binding, extracellular matrix binding, platelet-derived growth factor binding, calcium ion binding, and cell adhesion molecule binding. In the treated groups (Figures 2F–H), compared with the M group, most of the BPs, CCs, and MFs related to DEGs which were affected by baicalein; especially the high-dose baicalein and silymarin were overlapped (Supplementary Table S17). Figures 2E–H show KEGG pathway enrichment analysis of DEGs. The results indicated that baicalein exerting its protective effects against NAFLD was closely related to steroid biosynthesis, fatty acid degradation, the PPAR signaling pathway, peroxisome, tryptophan metabolism, ABC transporters, propanoate metabolism, valine, leucine and isoleucine degradation, 2-Oxocarboxylic acid metabolism, fatty acid metabolism, complement and coagulation cascades, ECM–receptor interaction, protein digestion and absorption, etc.

To verify the results of transcriptomic analysis, the expression of overlapped DEGs from three comparisons (P vs. M, L vs. M, and H vs. M) were detected by real-time PCR (Figure 2I). The expressions of *Apoa4*, *Pla2g12a*, *Elovl7*, *Slc27a4*, *Hilpda*, *Fabp4*, and *Vldlr* were all increased in the M group and reduced by the treatment of silymarin or baicalein. In contrast, the levels of *Gpld1* and *Apom* mRNA were decreased in the M group and positively regulated by silymarin or baicalein. The pathways associated with these genes were listed in Supplementary Table S1. Gene annotation, GO, and KEGG pathway analysis revealed that lipid metabolism–related pathways were significantly affected by baicalein.

### Baicalein Changes Gut Microbiota in Non-Alcoholic Fatty Liver Disease Mice

**FIGURE 3:**
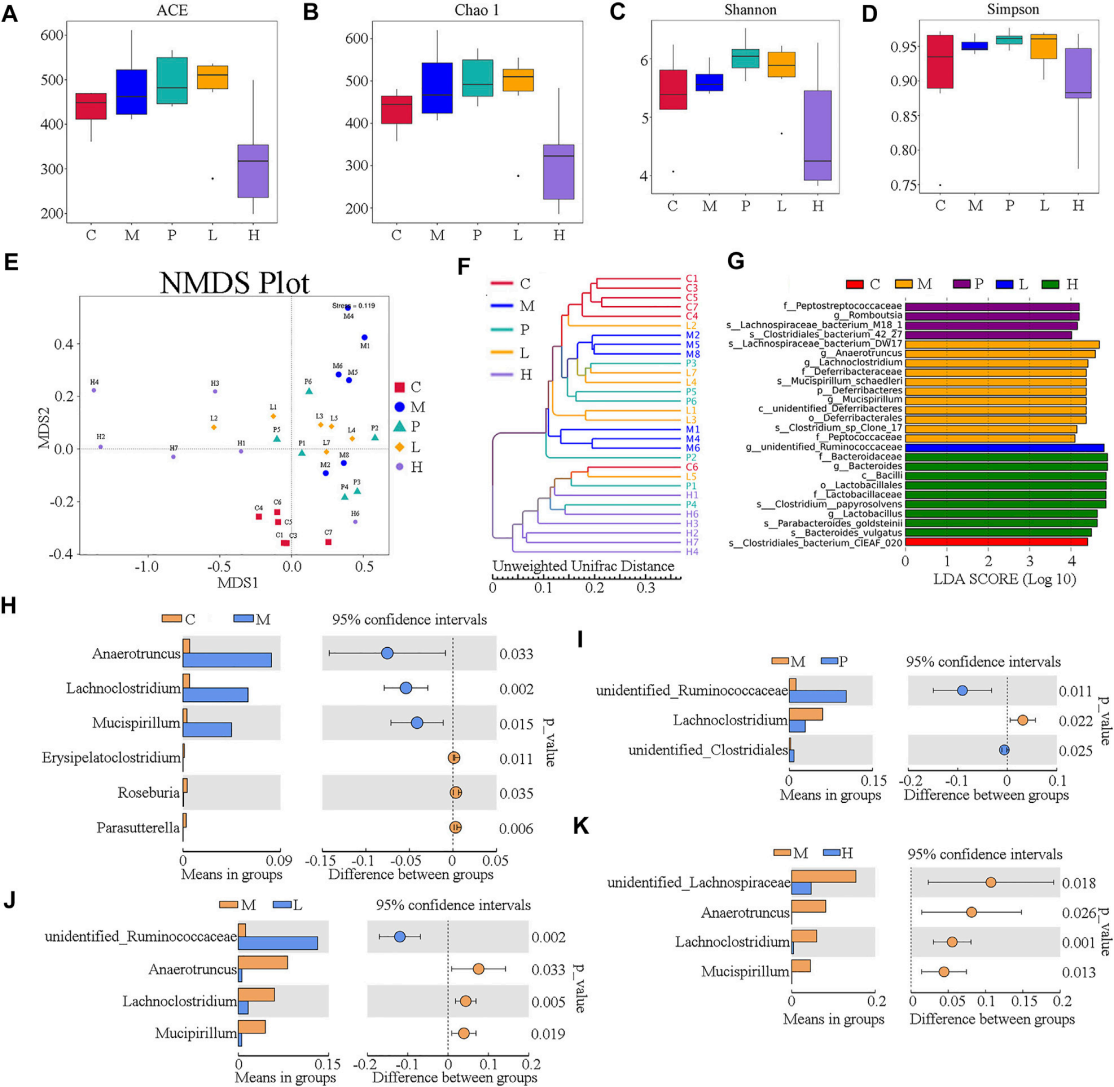
Overall structure of the gut microbiota was modulated after treatment with baicalein (*n* = 6). The α-diversity indexes, including ACE, Chao1, Shannon, and Simpson, are displayed in **(A–D)**, respectively. **(E)** UniFrac distance-based non-metric multidimensional scaling (NMDS). **(F)** UniFrac distance-based unweighted pair-group method with arithmetic means (UPGMA) analysis. **(G)** The score of the linear discriminant analysis (LDA) analysis. **(H)** STAMP variance analysis are displayed of M vs. C **(H)**, P vs. M **(I,C)**, L vs. M **(J)**, and H vs. M **(K)**.

The gut microbiome was assessed by 16S rRNA amplicon gene sequencing on an IonS5TMXL platform using specimen colonic luminal content samples of mice from C, M, P, L, and H groups. The α-diversity indexes including ACE, Chao1, Shannon, and Simpson were used to determine the ecological diversity within the microbial community. The ACE index reflecting the number of OTUs were 483.12 ± 78.15, 473.83 ± 98.86, and 318.11 ±109.97 in M, L, and H groups, respectively (Figure 3A). The Chao1 indexes reflecting community richness were 489.72 ± 85.24, 474.84 ± 102.03, and 309.88 ± 112.08 in M, L, and H groups, respectively (Figure 3B). The Shannon indexes indicating both the species richness and evenness were 5.63 ± 0.24, 5.75 ± 0.55, and 4.71 ± 1.06 in M, L, and H groups, respectively (Figure 3C). Simpson indexes reflecting community evenness were 0.95 ± 0.01, 0.95 ± 0.03, and 0.89 ± 0.07 in M, L, and H groups, respectively (Figure 3D). β-diversity analysis was used to investigate the overall community structure. As shown in PCoA (Supplementary Figure S4A) and NMDS (Figure 3E), the M group was separated from the C group significantly, and the baicalein-treated groups (L and H) were separated from the M group; especially H groups were isolated from the M group completely. In line with these results, unweighted UPGMA indicated that significant separation appeared between the C, M, P, L, and H groups (Figure 3F). These analyses confirmed the effect of baicalein on the microbiome structure remodeling in NAFLD mice.

To investigate the dominant microorganisms in response to the baicalein treatment, we analyzed the relative abundance in the phylum (Supplementary Figure S4B) and genus level (Supplementary Figure S4C) of each group and the species with significantly different abundances in the different groups using the linear discriminant analysis (LDA) effect size (LEfSe) method (Supplementary Figure S4D; Figure 3G). In the comparison of the baicalein-treated groups and the NAFLD model group at different taxonomic levels (Supplementary Figure S4D), the successive circles corresponding to five phylogenetic levels (phylum, class, family, class, and genus) indicated that the microbiota belonging to the *Deferribacteraceae* family, *Deferribacteraceae* order, unidentified_*Deferribacteres*, and *Peptococcaceae* family were enriched in the mice of the M group, whereas those belonging to the *Bacteroidaceae* family, *Lactobacillaceae* family, *Lactobacillales* order, and *Bacilli* class were enriched in the H group. In the P group, the *Peptostreptococcaceae* family was enriched. These results were also described in the LEfSe bar (Figure 3G). Also, the microbiota belonging to the Clostridiales_bacterium_CIEAF_020 (the C group) and the unidentified_*Ruminococcaceae* genus (the L group) were found to be enriched (Figure 3G). Notably, there were four different bacterial genera between the M group and the L or H group (Figures 3J,K), of which three were the same (*Anaerotruncus*, *Lachnoclostridium*, and *Mucispirillum*). Besides, the abundance of *Lachnoclostridium* was also increased in P group (Figure 3I). Interestingly, these three genera significantly increased in the M group compared with the C group (Figure 3H) were all drastically decreased following baicalein treatment. These findings indicated an appreciable capability of the baicalein to recover the gut microbiota profile altered by the high-fat diet.

To reveal whether functional genes change with the structure of the gut microbiota, Tax4fun was employed to predict the bacterial functions of the members among different groups based on KEGG Orthology (KO) terms. According to the functional annotation and abundance information of samples in the database, the top 35 metabolic pathways, which were level 3 KEGG pathways, were selected to establish the heat map (Supplementary Figure S4E). The abundances of 10 unique pathways (K01955, K03088, K00936, K01153, K03046, K02004, K02003, K09687, K02470, and K02337) were higher in the M group than in the C group, while the remaining 25 pathways were lower, as determined by Student’s *t*-test (*p*-value < 0.05). However, silymarin and low and high concentration of baicalein could restore the abundances of five pathways (K02004, K01153, K02337, K03046, and K07497), while the abundances of 12 pathways, including K03088, K03763, K03737, K04759, K06147, K02529, K03798, K03657, K02026, K02027, K02025, and K03406, were restored by silymarin or low concentration of baicalein.

### Variations of Metabolomic Profiling in Liver of Non-Alcoholic Fatty Liver Disease Mice

**FIGURE 4:**
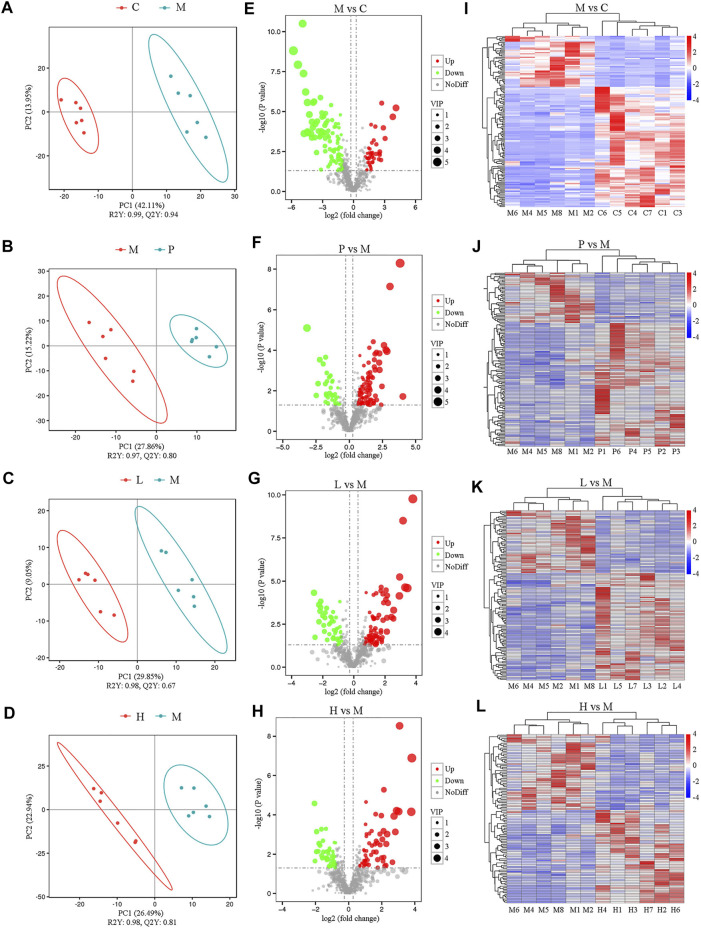
Metabolomic analysis among five groups under the positive ion mode (*n* = 6). The separation of two groups was accessed by partial least square-discriminate analysis (PLS-DA) [**(A)** M vs. C; **(B)** P vs. M; **(C)** L vs. M; and **(D)** H vs. M]. Volcano plot indicates the differentially expressed metabolites between the groups [**(E)** M vs. C; **(F)** P vs. M; **(G)** L vs. M; and **(H)** H vs. M]. Heat map shows the differential expression of metabolites between groups [**(I)** M vs. C; **(J)** P vs. M; **(K)** L vs. M; and **(L)** H vs. M].

To investigate the effects of baicalein on the metabolic profiling in NAFLD mice, LC-MS was used to analyze the extract from liver tissue of each group. Under the positive ion mode, data of 845 metabolites were used in partial least squares discriminant analysis (PLS-DA). As shown in Figure 4A, the M group and the C group could be clearly separated in the *x*-axis direction. The model quality was determined by parameters R2Y and Q2Y (R2Y = 0.99, Q2Y = 0.94). In addition, under the negative analysis ion mode, the PLS-DA score plot also showed a clear separation between the M and C groups (R2Y = 1.0 and Q2Y = 0.95) (Supplementary Figure S5A). Similarly, the significant separation was also observed between the M group and the P, L, or H group (Figures 4B–D). Besides, under the negative ion mode, the PLS-DA score plot also showed a clear separation between the M group and treatment groups (P, L, and H groups) (Supplementary Figures S5B–D).Therefore, the PLS-DA model could sufficiently explain the variance between these two groups.

Furthermore, significantly differential metabolites were identified according to the following criteria: PLS-DA VIP (variable importance in the projection) > 1, fold change > 1.5 and FDR < 0.05. We identified 148 metabolites that were differentially expressed in the M group as compared with the C group under the positive ion mode (Supplementary Table S18). Among significantly differential metabolites, 44 metabolites (Urobilinogen, 13-deoxytedanolide, 8-Azaadenosine, UROBILIN, Patidegib, p-Hydroxyketorolac, Bicyclomycin, S-[(1Z)-N-Hydroxy-5-(methylsulfanyl)pentanimidoyl]cysteine, Perflubron, Meptin, and so forth) were upregulated, while the remaining compounds were downregulated in the M group (Supplementary Table S18). Compared with the M group, there were 88, 77, and 58 metabolites upregulated and 36, 45, and 47 metabolites downregulated in the P (Supplementary Table S19), L (Supplementary Table S20), and H (Supplementary Table S21) groups, respectively. All these metabolites were included in the multivariate analysis, as shown in volcano plots (Figures 4E–H) and heatmaps (Figures 4I–L). Similar results were also observed under the negative analysis ion mode (Supplementary Figures S5E–L). These results suggested that baicalein treatment led to significant metabolic alterations in mouse liver.

KEGG pathway enrichment analysis was performed to determine the effect of baicalein on related pathways in NAFLD mice (Supplementary Figure S6). Compared with the C group, there were 11 related metabolic pathways under positive ion mode, and 13 related metabolic pathways in negative mode in the M group. The results indicated that primary bile acid biosynthesis (*p* = 3.98 × 10^–2^) and alpha-Linolenic acid metabolism (*p* = 3.98 × 10^–2^) were both downregulated in the M group and restored by silymarin (P group, *p* = 5.73 × 10^–2^) and baicalein (L group, *p* = 3.21 × 10^–2^). Additionally, silymarin and low concentration of baicalein might modulate 2-Oxocarboxylic acid metabolism (*p* = 5.73 × 10^–2^ in the P group and *p* = 3.21 × 10^–2^ in the L group). Interestingly, high concentration of baicalein could affect 2-Oxocarboxylic acid metabolism (*p* = 1.91 × 10^–2^ in positive ion mode), Pantothenate and CoA biosynthesis (*p* = 2.41 × 10^–2^ in negative ion mode), and bile secretion (*p* = 4.96 × 10^–2^ in negative ion mode) (Supplementary Figures S6D,H). Taken together, baicalein was closely associated with altered alpha-Linolenic acid, 2-Oxocarboxylic acid, Pantothenate and CoA biosynthesis, and bile secretion.

### Integrated Analysis of the Mechanism of Baicalein Treated Mice From Metabolomic and Transcriptomic Data

**FIGURE 5:**
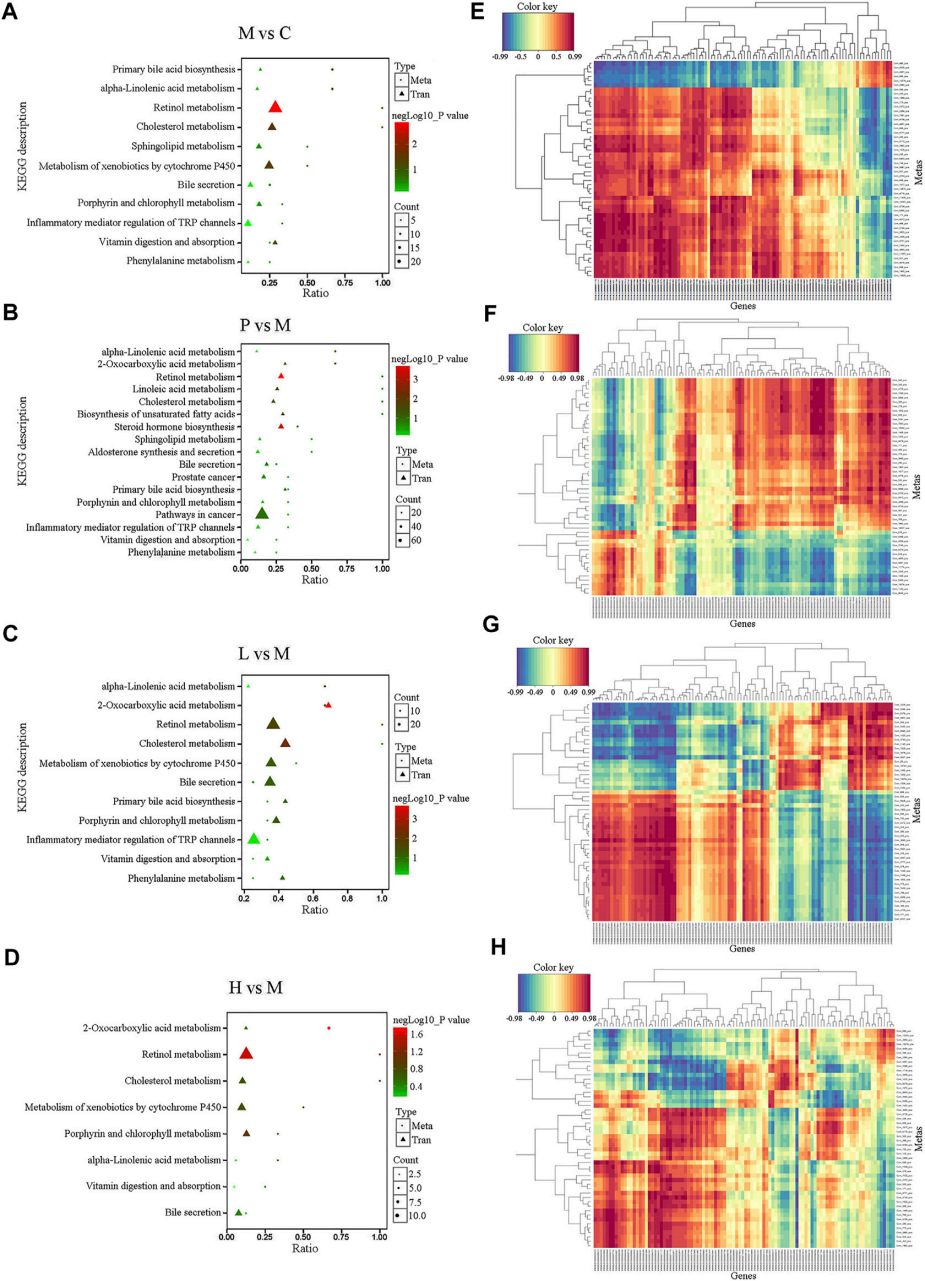
Integrated analysis of the metabolomic and transcriptomic data under the positive ion mode. **(A–D)** Integrated altered metabolic pathways according to the metabolomic and transcriptomic data. The horizontal axis shows the ratio of differential expressed genes and the vertical axis indicated the pathway name [**(A)** M vs. C; **(B)** P vs. M; **(C)** L vs. M; and **(D)** H vs. M]. **(E–H)** Integrated analysis using metabolomic and transcriptomic data. The horizontal axis shows the clustering of differential expressed transcripts and the vertical axis indicated the clustering of differential expressed metabolites. The red depth represents the strength of the positive correlation. The blue depth represents the negative correlation [**(E)** M vs. C; **(F)** P vs. M; **(G)** L vs. M; and **(H)** H vs. M].

To explore the potential relationship between DEGs and differential metabolites in the liver, metabolomic and transcriptomic data were integrated. Compared with the C group, a total of 12 pathways were listed under the negative ion mode (as shown in Supplementary Figure S7A, such as Porphyrin and chlorophyll metabolism, Platelet activation, Drug metabolism—cytochrome P450, Protein digestion and absorption, Cholesterol metabolism, Steroid hormone biosynthesis, and so forth) and 11 ones (as shown in Figure 5A, such as Primary bile acid biosynthesis, alpha−Linolenic acid metabolism, Retinol metabolism, and Cholesterol metabolism) in the positive ion mode in the M group. Compared with the M group, the significantly differential metabolites in the P (Figure 5B), L (Figure 5C), and H (Figure 5D) groups were involved in 17, 11, and 8 pathways, respectively, under the positive ion mode, while there were 8 (Supplementary Figure S7B), 13 (Supplementary Figure S7C), and 13 (Supplementary Figure S7D) pathways, respectively, under the negative ion mode. Among them, steroid hormone biosynthesis, cholesterol metabolism, primary bile acid biosynthesis, bile secretion, and drug metabolism-cytochrome P450 might play an important role in the process of baicalein and silymarin ameliorating NAFLD symptoms. Besides, low concentration of baicalein affected three pathways (including Serotonergic synapse, Protein digestion, and absorption and Platelet activation), which were also altered in the M group. Notably, low and high dose of baicalein both affected fatty acid biosynthesis, and high dose of baicalein also modulated some different pathways, such as fatty acid degradation, fat digestion, and absorption, pantothenate and CoA biosynthesis, vitamin digestion and absorption, beta-alanine metabolism, ferroptosis, starch and sucrose metabolism, cholinergic synapse, and retrograde endocannabinoid signaling. There is also a heat map of integrated analysis between differential metabolite expression patterns and transcriptomics under the positive ion mode (Figures 5E–H) and negative ion mode (Supplementary Figures S7E–H). Taken together, baicalein mainly targeted the pathways asociated with biosynthesis and secrection of bile acid, such as cholesterol metabolism and steroid hormone biosynthesis and the pathways related to lipid metabolism including fatty acid degradation, fat digestion and absorption, and so on. These were consistent with the results of real-time PCR described above (Figure 2I). For example, *Apoa4* plays a role in fat digestion and absorption, cholesterol metabolism, and atherosclerosis; *Pla2g12a* functions in Glycerophospholipid metabolism, ether lipid metabolism, arachidonic acid metabolism, linoleic acid metabolism, and fat digestion and absorption; *Elovl7*, *Slc27a4*, *Hilpda*, and *Fabp4* are all involved in lipid metabolism (Supplementary Table S1).

### Association of the Non-Alcoholic Fatty Liver Disease–Induced Gut Microbial Dysbiosis With Dysregulation Metabolites

**FIGURE 6:**
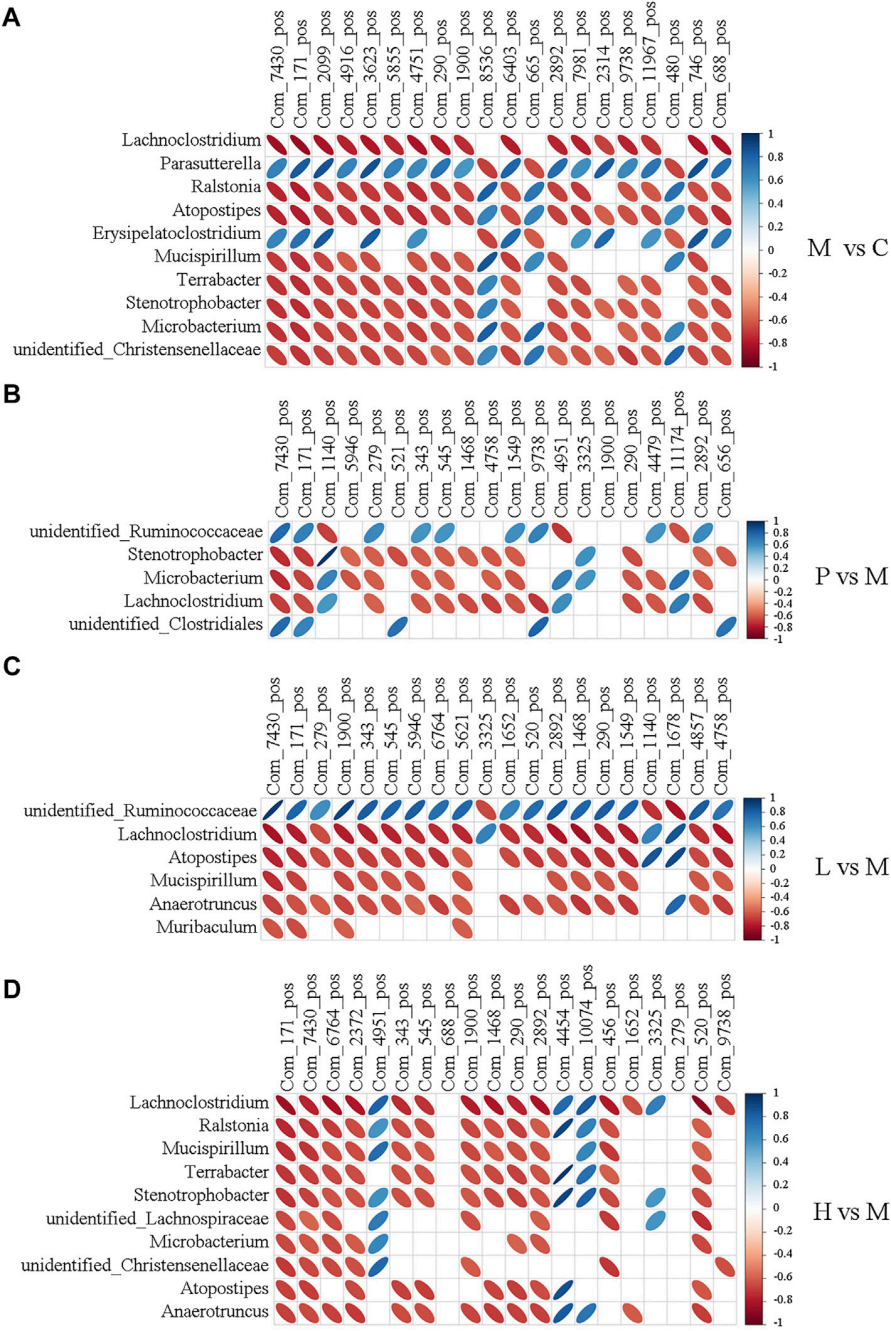
Heatmap describing the correlation analysis of relative abundance of gut microbiota in the genus level and liver metabolite levels under the positive ion mode. **(A)** M vs. C; **(B)** P vs. M; **(C)** L vs. M; and **(D)** H vs. M. The horizontal axis shows the top 20 of the differential metabolites in the comparison pair, the left vertical axis shows the differential bacteria, and the right means the correlation coefficient. Blue indicates positive correlation and red indicates negative correlation.

To further explore the potential association between gut microbiota and liver metabolome in NAFLD mice, the top 20 differentially expressed metabolites and the top 10 altered microbial genera between two groups were analyzed by Pearson’s correlation analysis. As Figure 6 displayed, the typical metabolites of physiological function were highly linked to specific gut bacteria by calculating Pearson’s correlation coefficient. For instance, compared with the C group, the abundance of *Lachnoclostridium* showed a significantly negative correlation with 17 metabolites except Patidegib, urobilinogen, and 13-deoxytedanolide in the M group. 2-Hydroxyimipramine and L-Ergothioneine, which decreased to less than 4% and 2% in the C group, were positively associated with *Parasutterella* (*r* = 0.665 and 0.822) and *Erysipelatoclostridium* (*r* = 0.657 and 0.739) and negatively correlated with *Lachnoclostridium* (*r* = −0.83 and −0.857), *Microbacterium* (*r* = −0.718 and −0.736), *Ralstonia* (*r* = −0.755 and −0.774), *Atopostipes* (*r* = −0.752 and −0.773), *Mucispirillum* (*r* = −0.697 and −0.72), *Terrabacter* (*r* = −0.717 and −0.738), *Stenotrophobater* (*r* = −0.716 and −0.739), and unidentified_*Christensenellaceae* (*r* = −0.682 and −0.703). Notably, baicalein treatment clearly regulated the level of 2-Hydroxyimipramine and L-Ergothioneine. They were negatively associated with *Lachnoclostridium*, *Atopostipes*, *Mucispirillum*, *Anaerotruncus,* and *Muribaculum* except unidentified_*Ruminococcaceae* in the L group. However, they were negatively associated with *Lachnoclostridium*, *Ralstonia*, *Mucispirillum*, *Terrabacter*, *Stenotrophobacter*, unidentified_*Lachnospiraceae*, *Microbacterium*, unidentified_*Christensenellaceae*, *Atopostipes,* and *Anaerotruncus* in the H group. The abundances of *Lachnoclostridium*, *Atopostipes*, *Mucispirillum,* and *Anaerotruncus* were all decreased in the L and H groups (Figures 3J,K), and the first three were increased in the M group (Figure 3H). The results were also observed under the negative ion mode (Supplementary Figure S8). L-Ergothioneine, which is a metabolite with anti-oxidant activity, protects the liver against lipid peroxidation and is also involved in histidine metabolism. Our findings indicated that the abundance of L-Ergothioneine was affected by some microbial genera which were regulated by baicalein. Therefore, baicalein affected lipid metabolism in the liver by regulating the ecological structure of gut microbiota in NAFLD mice.

## Discussion

The pathogenesis of NAFLD is complex. Nowadays, the “multiple-hit model” is the theory widely accepted. The metabolic dysfunction induced by the genetic defects, environmental factors, and abnormal interaction of the organs and tissues is identified as the direct cause of NAFLD. However, fat accumulation in the liver seems to be the “first hits” (Fang et al., 2018). Triglycerides derived from the esterification of glycerol and free fatty acids (FFAs) are the main form of fat accumulated in the liver (Musso et al., 2013). Insulin resistance (IR) is a critical stage and risk factor in the progression of NAFLD. IR promotes liver lipid synthesis and inhibits liver fatty acid β-oxidation and lipolysis of fat. Consequently, lipids accumulate in the liver, and finally, hepatocyte fatty lesions occur (Stols-Gonçalves et al., 2019).

However, the available therapeutic options are very limited so far. Currently, the main treatment strategies against NAFLD include lifestyle change (diet and exercise), anti-oxidant treatment (such as vitamin 4), and regulation of body metabolism and lipid regulation. Silymarin, which is a complex mixture of flavonolignan isomers, namely, silybin, isosilybin, silydianin, and silychristin, from *Silybum marianum* (milk thistle), has been proved to be effective in treating liver diseases including NAFLD. Silymarin has been widely used in the treatment of acute or chronic hepatitis (Tighe et al., 2020). Baicalein is also a natural flavonoid extracted from plants. Growing evidence has indicated the anti-oxidant, anti-inflammatory, and antitumor activities of baicalein. In this study, we proved that baicalein significantly protected liver function against HFD-induced NAFLD (Figure 1). In terms of therapeutic effect, baicalein is slightly stronger than silymarin.

Gut microbiota, including bacteria, fungi, parasites, and viruses, inhabits the gastrointestinal tract. Increasing evidence indicated that gut microbiota plays an important role in maintaining the integrity of the intestinal epithelium, defense against pathogens, regulating the host immunity, harvesting energy, and regulating metabolism. In healthy adults, *Firmicutes*, *Bacteroidetes*, *Actinobacteria*, and *Proteobacteria* are the top 4 phyla in abundance. The abundance of these bacterial phyla is usually related to some pathologic conditions, including NAFLD. In NAFLD, the microbial diversity is usually decreased, with the increased relative abundance of species of the *Proteobacteria* and *Bacteroidetes* phyla and the *Enterobacteriaceae* family and the *Escherichia* genera and reduced relative abundance of the *Firmicutes* phylum and the *Prevotellaceae* family. Consequently, lipopolysaccharide (LPS) translocation is increased, short-chain fatty acids (SCFA) produced by gut microbiota are decreased, and the production of endogenous ethanol is increased, and then inflammations occur. Le Roy and colleagues believed that intestinal microbiota allows the transfer of disease phenotype and liver steatosis by conducting gut microbiota transplant experiments (Le Roy et al., 2013). Alterations of gut microbiota composition affect the expression of intestinal and hepatic genes related to the metabolic process and the onset of inflammation (Membrez et al., 2008). In this study, we identified that the composition of gut microbiota was extremely changed in NAFLD mice compared with the control group, and the treatment of baicalein as well as silymarin could restore the abundance of some genera, especially the abundances of *Anaerotruncus*, *Lachnoclostridium*, and *Mucispirillum* that were decreased under the treatment of silymarin and low or high dose of baicalein (Figure 3). The abundance of *Anaerotruncus* and *Mucispirillum* were proved increased in high-fat/high-cholesterol–fed mice and related to NAFLD-associated hepatocellular carcinoma development (Zhang et al., 2021). The relative abundance of *Lachnoclostridium* associated with inflammation-mediated obesity (Rondina et al., 2013) was remarkably higher in the HFD group than in the control group and positively correlated with the fructose levels in feces (Jo et al., 2021). Therefore, gut microbiota seems to be the potential therapeutic target of baicalein against NAFLD.

Recently, gut microbiota is considered to be an important “organ” of the body, which can affect a variety of physiological processes. Dao et al. (2021) believed that gut microbiota altered by high-fat diet could modulate the retinal transcriptome, and they proposed a diet–microbiome–retina axis to reveal how diet affects the pathogenesis and severity of retinal diseases. Another report showed that gut microbiota from young donors could reprogram the circadian clock of the lacrimal gland by transcriptomic analysis (Jiao et al., 2021). A study on NAFLD demonstrated that appropriate WLT supplementation could regulate gut microbial composition, reduce intestinal permeability and liver inflammation, and then prevent NAFLD (Chen et al., 2021). Therefore, gut microbiota can affect the gene expression of a variety of tissues, including the liver, and then play a regulatory role in human diseases. In this study, we found that compared with the control mice, the gut microbial composition of NAFLD mice was changed significantly, and baicalein could restore the relative abundance of some bacteria. Based on the analysis of liver transcriptome, we found that many transcripts changed by baicalein overlapped with those changed by silymarin. Silymarin is considered a natural drug that can effectively resist liver inflammation and is used clinically in the treatment of NAFLD. The expressions of several genes related to lipid metabolism were verified by real-time PCR to confirm the alteration of liver metabolites (Figure 2I). For example, *Apoa4*, *Pla2g12a*, and *Slc27a4*, which all promote fat digestion and absorption, were negatively regulated by silymarin and baicalein. *Gpld1* and *Apom*, which are both the positive regulators of lipid metabolism, were increased by baicalein. Therefore, compared with silymarin, we believe that baicalein has a similar function of alleviating NAFLD, and there might be a crosstalk in the mechanism.

Gut microbiota also regulate the choline metabolism and then affect the accumulation level of hepatic triglycerides (Panasevich et al., 2017). The bile acid-farnesoid X receptor (FXR) may be an important participant in the process of gut microbiota modulating body weight and hepatic steatosis in mice (Parséus et al., 2017). The fasting-induced adipocyte factor (FIAF) reduced by gut microbiota was proved to exacerbate the accumulation of hepatic triglycerides (Roopchand et al., 2015). Besides, gut microbiota–mediated excess of short-chain fatty acids (SCFAs) in the liver reduces the activity of adenosine-monophosphate activated protein kinase (AMPK), leading to the accumulation of hepatic free fatty acids (Wang et al., 2020). With the analysis of liver metabolome, we found that primary bile acid biosynthesis and alpha-Linolenic acid metabolism were downregulated in NAFLD mice and restored by baicalein and silymarin. Previous studies have proved that fatty acids, bile acids, and amino acids are involved in the development of hyperlipidemia (Li et al., 2018; Liu et al., 2019). The pathogenesis of NAFLD is due to the accumulation of excessive lipid in hepatocytes, which is induced by the transfer of excessive NEFA from adipose tissue to the liver, the increase in fat synthesis, the reduction of fatty acid β-oxidation, and bile acid excretion (Tang et al., 2019). Bile acids are produced from cholesterol in hepatocytes and regulate insulin secretion and glycolipid metabolism. Guo et al. (2020) found that Ganoderic acid A ameliorated hyperlipidemia and gut microbiota dysbiosis in HFD-induced hyperlipidemic mice, and bile acid metabolism was positively regulated by Ganoderic acid A. Alpha-linolenic acid is a metabolite with antioxidant activity. Lianqun et al. found that alpha-linolenic acid metabolism was significantly downregulated in hyperlipidemia model mice. After ginsenoside Rb1 intervention, gut microbiota of mice was remarkably changed and alpha-linolenic acid metabolism was clearly upregulated (Lianqun et al., 2021). In the research on human schizophrenia, Fan et al. (2022) discovered a significant correlation between serum differential metabolites and differential intestinal bacteria between the patients with schizophrenia and the healthy individuals, while the metabolism of anti-inflammatory metabolites (such as alpha-linolenic acid) might be the regulatory target of gut microbiota. Therefore, the primary bile acid biosynthesis and alpha-linolenic acid metabolism are closely related to lipid metabolism. In the integrative analysis of multi-omics, we found that there was a significant correlation between the relative abundance of altered intestinal bacterial genera and the levels of various metabolites. For example, the relative abundance of *Lachnoclostridium*, *Atopodipes*, *Mucispirillum*, and *Anaerotruncus* was significantly restored to the normal level in the baicalein treatment group and had a clear negative correlation with the level of some metabolites. For example, L-Ergothioneine was enriched in Histidine metabolism, and Traumatic acid was enriched in alpha-linolenic acid metabolism. Thus, baicalein intervention could significantly improve a considerable number of pathways, including the primary bile acid biosynthesis and alpha-linolenic acid metabolism, and the associated mechanisms might be the direct regulation by baicalein, indirect modulation by baicalein *via* gut microbiota, or the comprehensive effect of multiple mechanisms affected by baicalein.

In conclusion, baicalein may affect the microbial community structure in the intestine on the one hand and affect the expression of liver transcriptome through its own metabolism on the other hand and then affect the hepatic fatty acid metabolism against NAFLD. However, this study only focused on intestinal bacteria, and the role of intestinal fungi and other microorganisms in NAFLD is unknown. Moreover, the mechanism of how intestinal microorganisms respond to baicalein and then affect the expression of liver transcripts is unclear. Therefore, we will further investigate the intestinal fungi and other microorganisms in NAFLD mice and analyze the role of intestinal endothelial cells in the function of gut microbiota, hoping to uncover the molecular mechanism of baicalein in alleviating NAFLD.

## Data Availability

The datasets presented in this study can be found in online repositories. The names of the repository/repositories and accession number(s) can be found in the article/Supplementary Material.

## Supplementary Material

Supplementary Figure S1 Effects of baicalein on high-fat diet–induced hyperglycemia. **(A)** Scores of steatosis, inflammation and ballooning, and Liver NAS score. **(B)** Fasting blood glucose (mM); **(C)** Fastingserum insulin (mU/mL). Data are presented as mean ± SD (*n*=10). ***p* < 0.01, **p* < 0.05, compared with the C group; ##*p* < 0.01, #*p* < 0.05, compared with the M group.

Supplementary Figure S2 DEGs involved in C, M, P, L, and H groups (*n*=3). (A) The heatmap showing the DEGs involved in five groups. (B) The Venn diagram indicating the DEGs overlapped in three comparisons, M vs. P, M vs. L, and M vs. H.

Supplementary Figure S3 DEGs were enriched in the GO database (*n*=3). (A) M vs. C; (B) P vs. M; (C) L vs. M; (D) H vs. M).

Supplementary Figure S4 Community composition of gut microbiota was regulated after treatment with baicalein (*n*=6). **(A)** Principal coordinate analysis (PCoA) based on the unweighted UniFrac analysis calculated using QIIME software. Effects of baicalein on the composition of the microbiota at phylum **(B)** and genus **(C)** levels. **(D)** LefSe analysis based on the linear discriminant analysis (LDA). **(E)** Heatmap showing the specific annotated information of each KEGG orthologous group (KO) in the metabolic pathway.

Supplementary Figure S5 Metabolic alterations after baicalein treatment in the liver under the negative ion mode (*n*=6). Partial least squares discriminant analysis (PLS-DA) was used to show the separation between two groups, including M and C **(A)**, P and M **(B)**, L and M **(C),** and H and M **(D)**. The differential metabolites were displayed in the volcano plots [**(E)** M vs. C; **(F)** P vs. M; **(G)** L vs. M; **(H)** H vs. M] and heatmaps [**(I)** M vs. C; **(J)** P vs. M; **(K)** L vs. M; **(L)** H vs. M].

Supplementary Figure S6 KEGG pathway enrichment analysis under the positive and the negative ion mode (*n*=6). **(A–D)** showed the differential metabolites between M and C, P and M, L and M, and H and M, respectively, under the positive ion mode. **(E–H)** indicated the differential metabolites under the negative ion mode.

Supplementary Figure S7 Integrated analysis of the relationship between DEGs and differential metabolites in the liver under the negative ion mode. **(A–D)** Integrated altered metabolic pathways according to the metabolomic and transcriptomic data. The horizontal axis shows the ratio of differential expressed genes and the vertical axis indicated the pathway name [**(A)** M vs. C; **(B)** P vs. M; **(C)** L vs. M; **(D)** H vs. M]. **(E–H)** Integrated analysis using metabolomic and transcriptomic data. The horizontal axis shows the clustering of differential expressed transcripts and the vertical axis indicated the clustering of differential expressed metabolites. The red depth represents the strength of the positive correlation. The blue depth represents the negative correlation [**(E)** M vs. C; **(F)** P vs. M; **(G)** L vs. M; **(H)** H vs. M].

Supplementary Figure S8 Heatmap describing the correlation analysis of relative abundance of gut microbiota in the genus level and liver metabolite levels under the negative ion mode. **(A)** M vs. C; **(B)** P vs. M; **(C)** L vs. M; **(D)** H vs. M. The horizontal axis shows the top 20 of the differential metabolites in the comparison pair, the left vertical axis shows the differential bacteria, and the right means the correlation coefficient. Blue indicates positive correlation and red indicates negative correlation.

Supplementary Table S1 Genes detected by real-time PCR.

Supplementary Table S2 Number of DEGs between groups.

Supplementary Table S3 Transcripts upregulated in the M group compared with the C group.

Supplementary Table S4 Transcripts downregulated in the M group compared with the C group.

Supplementary Table S5 Transcripts upregulated in the P group compared with the M group.

Supplementary Table S6 Transcripts downregulated in the P group compared with the M group.

Supplementary Table S7 Transcripts restored to the normal level in the P group.

Supplementary Table S8 Transcripts upregulated in the L group compared with the M group.

Supplementary Table S9 Transcripts downregulated in the L group compared with the M group.

Supplementary Table S10 Transcripts restored to the normal level in the L group.

Supplementary Table S11 Transcripts upregulated in the H group compared with the M group.

Supplementary Table S12 Transcripts downregulated in the H group compared with the M group.

Supplementary Table S13 Transcripts restored to the normal level in the H group.

Supplementary Table S14 DEGs overlapped in the L group vs the M group and the H group vs the M group.

Supplementary Table S15 Transcripts positively regulated by baicalein.

Supplementary Table S16 Transcripts negatively regulated by baicalein.

Supplementary Table S17 GO pathways overlapped in P, L, and H groups.

Supplementary Table S18 Differential metabolites in the M and C groups under the positive ion model.

Supplementary Table S19 Differential metabolites in the P and M groups under the positive ion model.

Supplementary Table S20 Differential metabolites in the L and M groups under the positive ion model.

Supplementary Table S21 Differential metabolites in the H and M groups under the positive ion model.

Supplementary Table S22 Information of the top 20 correlated differential metabolites in M vs C.

Supplementary Table S23 Information of the top 20 correlated differential metabolites in P vs M.

Supplementary Table S24 Information of the top 20 correlated differential metabolites in L vs M.

Supplementary Table S25 Information of the top 20 correlated differential metabolites in H vs M.
