# Supplementary figures and images for: Multi-Omics Reveals Inhibitory Effect of Baicalein on Non-Alcoholic Fatty Liver Disease in Mice
Source: Front Pharmacol. 2022 Jun 15;13:925349. doi: 10.3389/fphar.2022.925349 (PMC9240231; doi:10.3389/fphar.2022.925349)

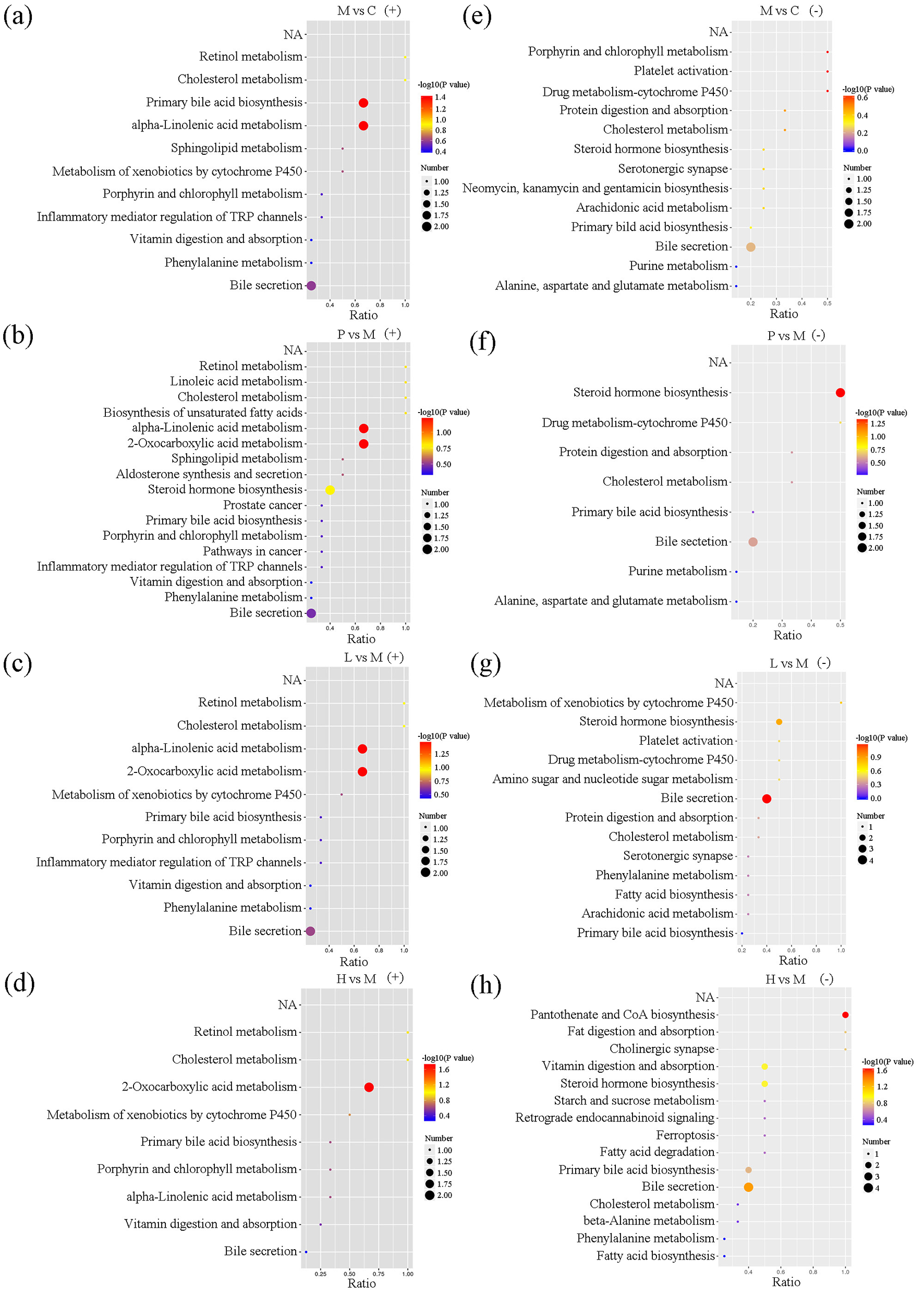

Supplement: Supplementary file 1 [file Image6.TIF]

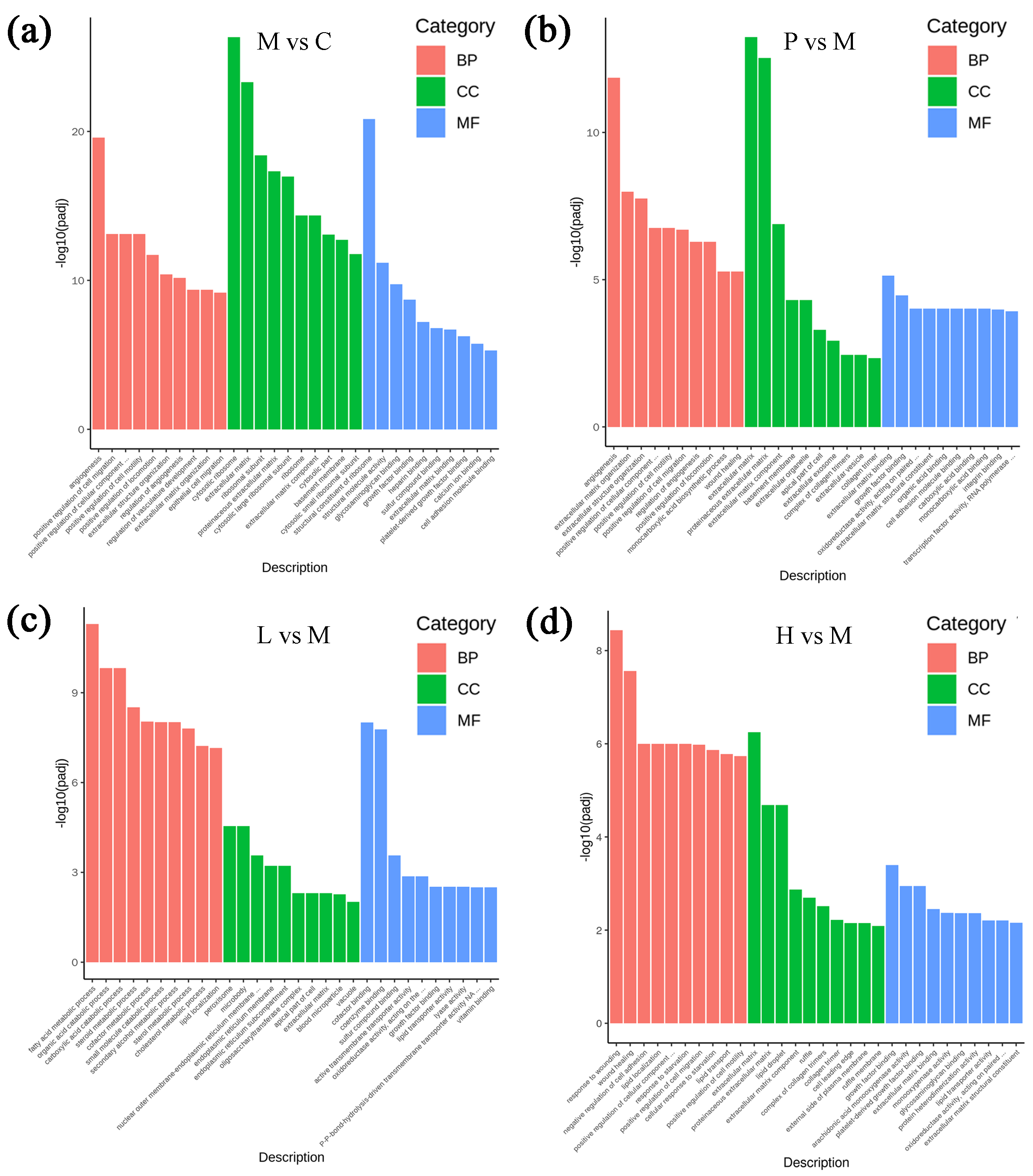

Supplement: Supplementary file 2 [file Image3.TIF]

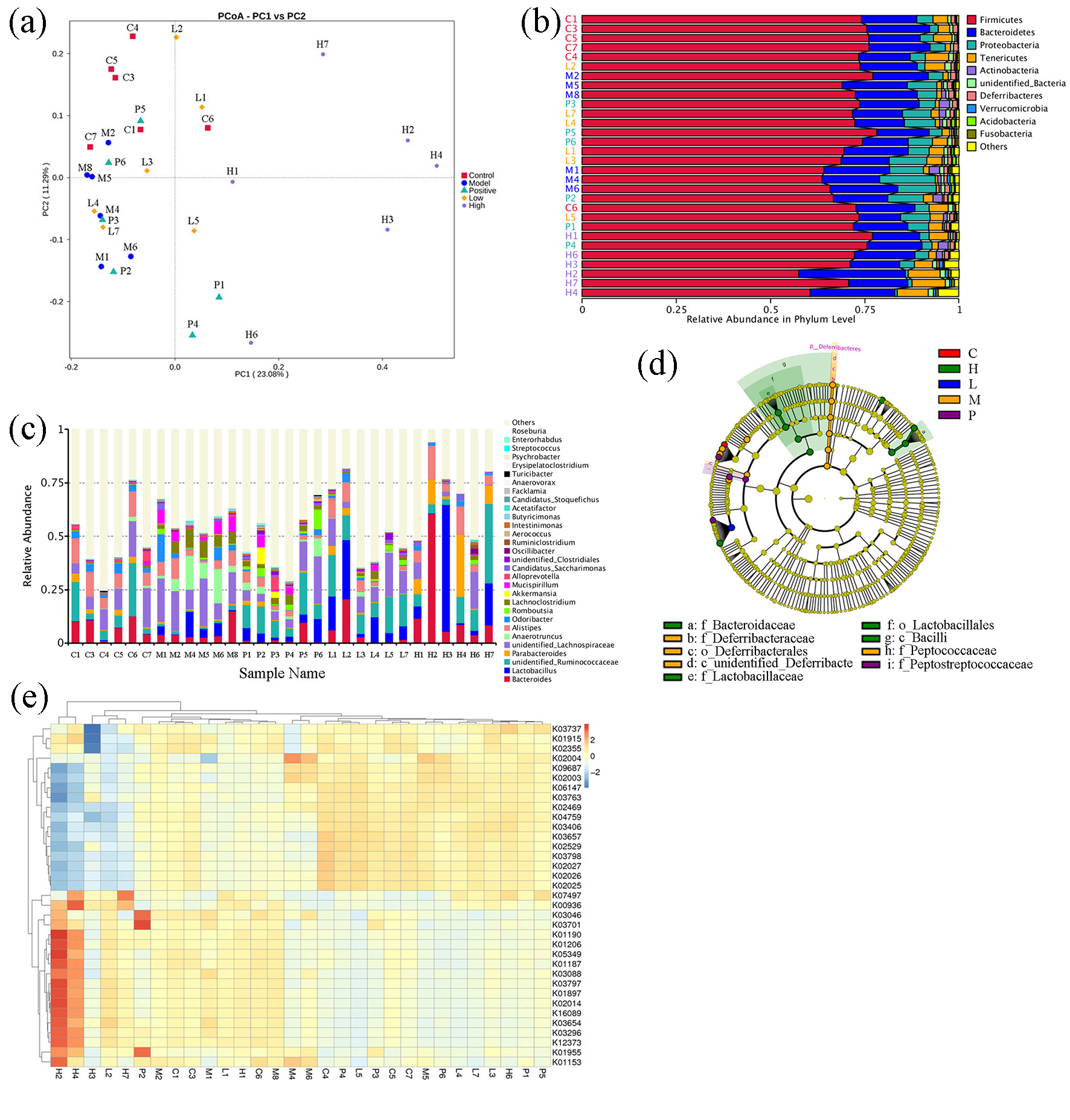

Supplement: Supplementary file 3 [file Image4.TIF]

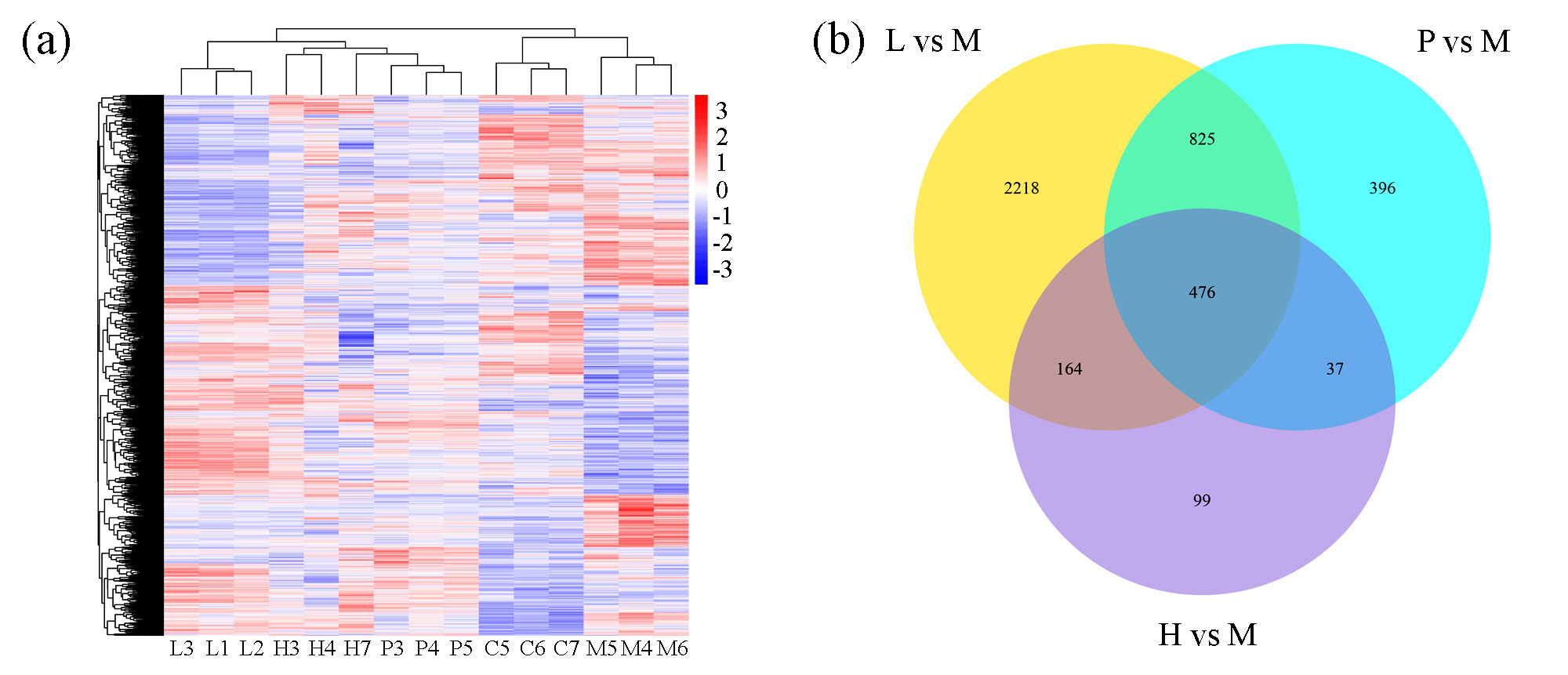

Supplement: Supplementary file 4 [file Image2.TIF]

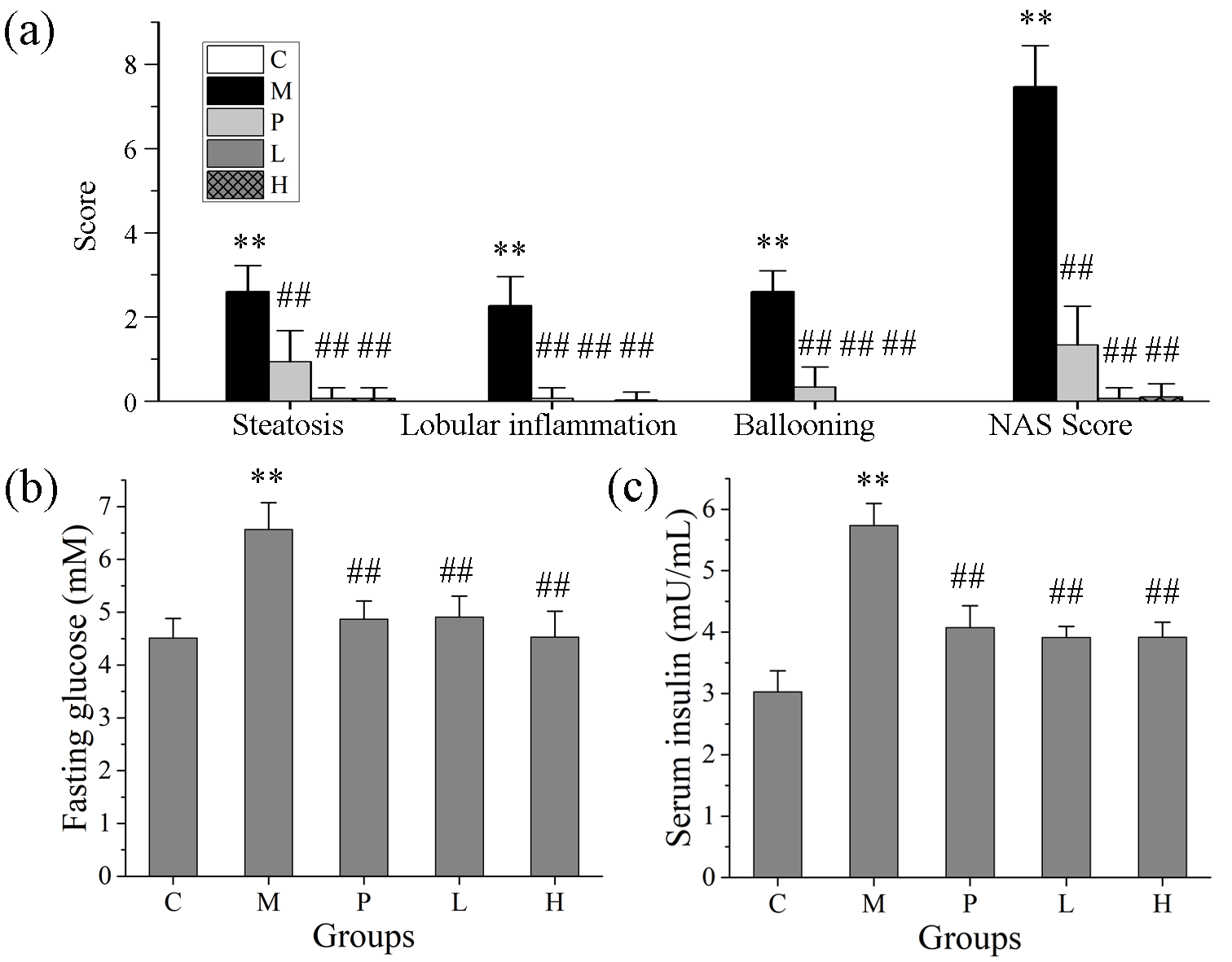

Supplement: Supplementary file 5 [file Image1.TIF]

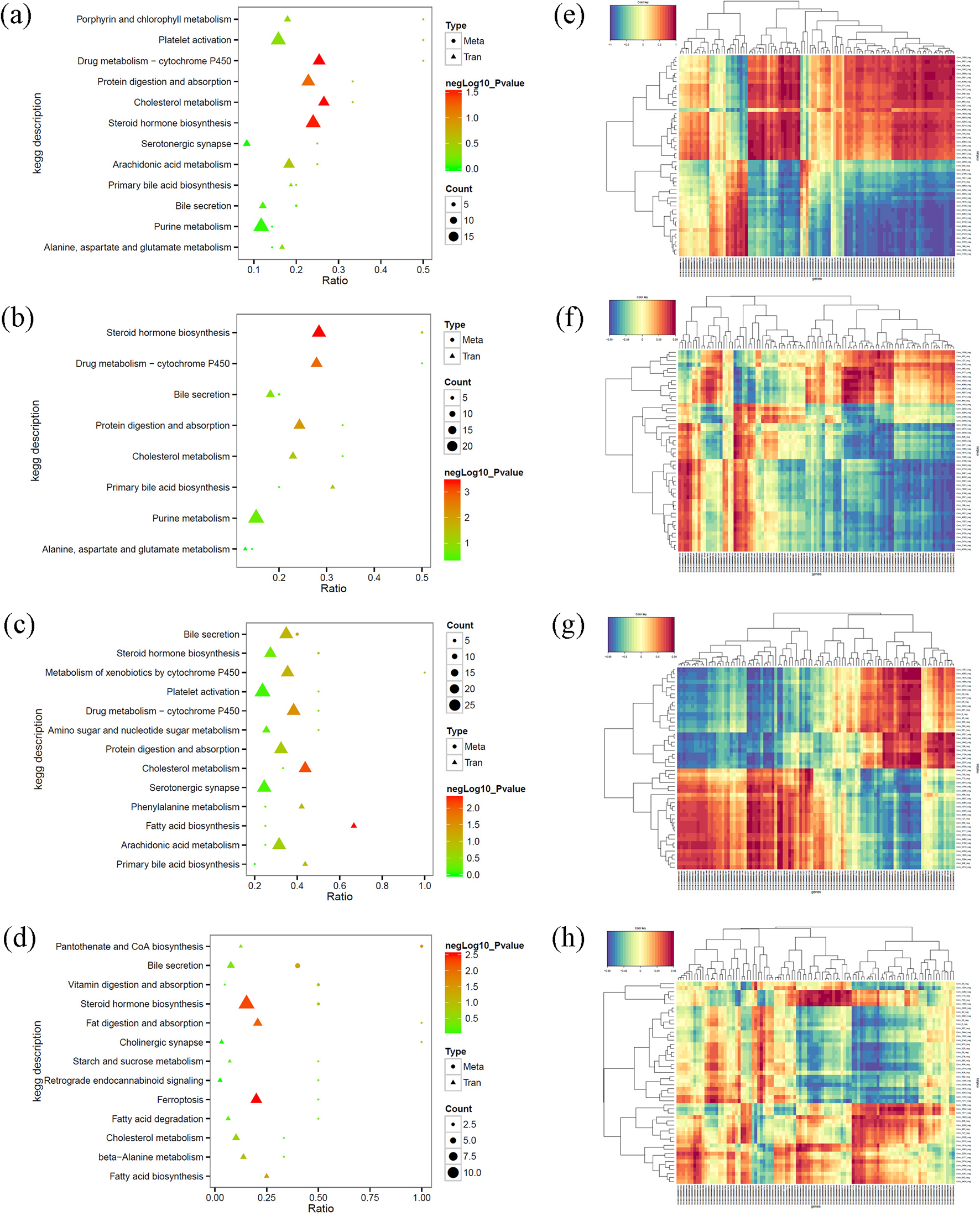

Supplement: Supplementary file 6 [file Image7.TIF]

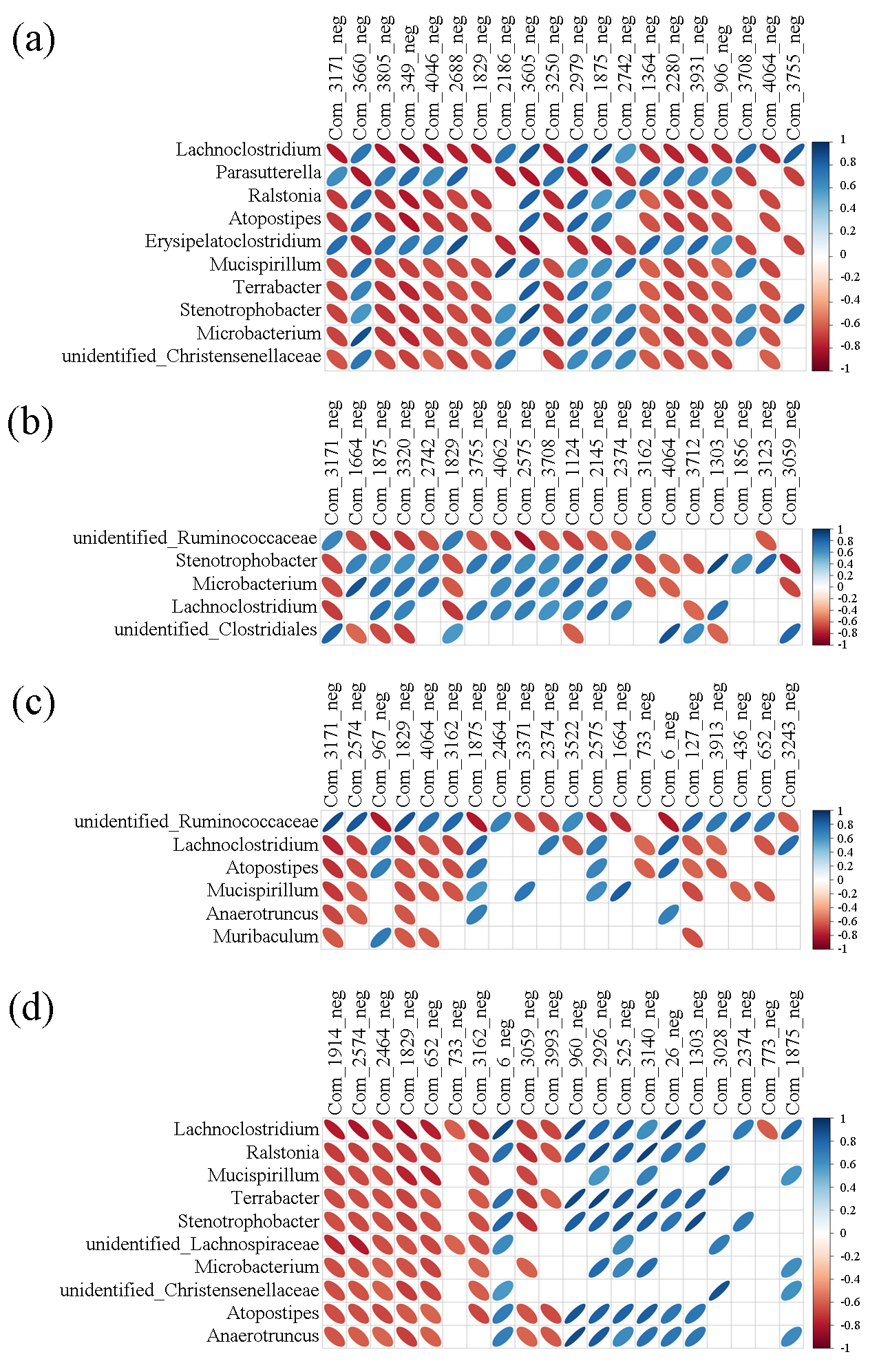

Supplement: Supplementary file 8 [file Image8.TIF]

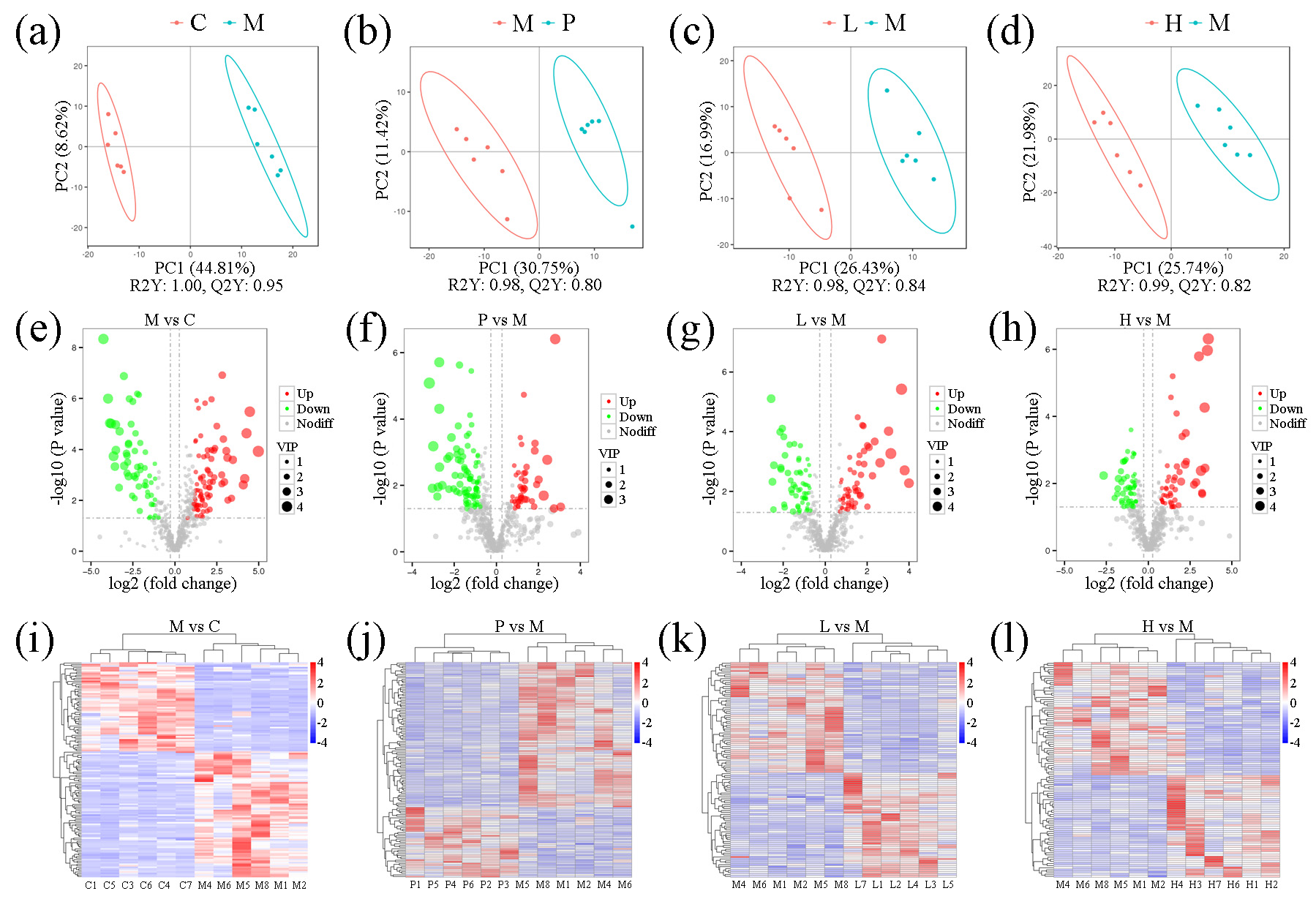

Supplement: Supplementary file 9 [file Image5.TIF]
